# Metastatic Colorectal Adenocarcinoma in a Bifid Ureter

**DOI:** 10.1089/cren.2018.0100

**Published:** 2019-03-18

**Authors:** Nikhil Gopal, Michael Stern, Neel H. Patel, Gerald Matthews, Majid Eshghi

**Affiliations:** Department of Urology, New York Medical College, Valhalla, New York.

**Keywords:** secondary ureteral metastasis, bifid ureter, ureteroscopy, colorectal adenocarcinoma

## Abstract

***Background:*** Secondary malignancies of the ureter are uncommon. We report the diagnosis and management of metastatic colon cancer to the bifurcation of a bifid ureter.

***Case Presentation:*** A 59-year-old man presented with diffuse metastasis with right hydronephrosis in both renal moieties of a partially duplicated system and an enhancing lesion within the proximal common ureter. Ureteral biopsy was positive for colorectal adenocarcinoma. The patient was subsequently started on palliative chemoradiation.

***Conclusion:*** The ureter is a rare location for hematogenous/lymphatic metastases. When a ureteral mass is present on imaging, ureteroscopy should be performed to characterize the extent of tumor and to rule out secondary malignancy.

## Introduction and Background

Although direct invasion of the ureter by primary malignancies (e.g., bladder) is not unusual, hematogenous or lymphatic metastasis to the ureter is rare, with only ∼400 cases described in the literature since 1909.^1^ We present a patient with colorectal adenocarcinoma metastasizing to the bifurcation of a bifid ureter, which, to our knowledge, has not been previously described.

## Case Presentation

### History

A 59-year-old man with hypertension and diabetes presented with progressively worsening back pain radiating down the right leg, right flank pain, nausea, and vomiting. He denied hematuria, dysuria, or any problems with urination. He also had an unintentional weight loss of 5 kg during the past 6 months.

### Diagnosis

**FIG. 1.**
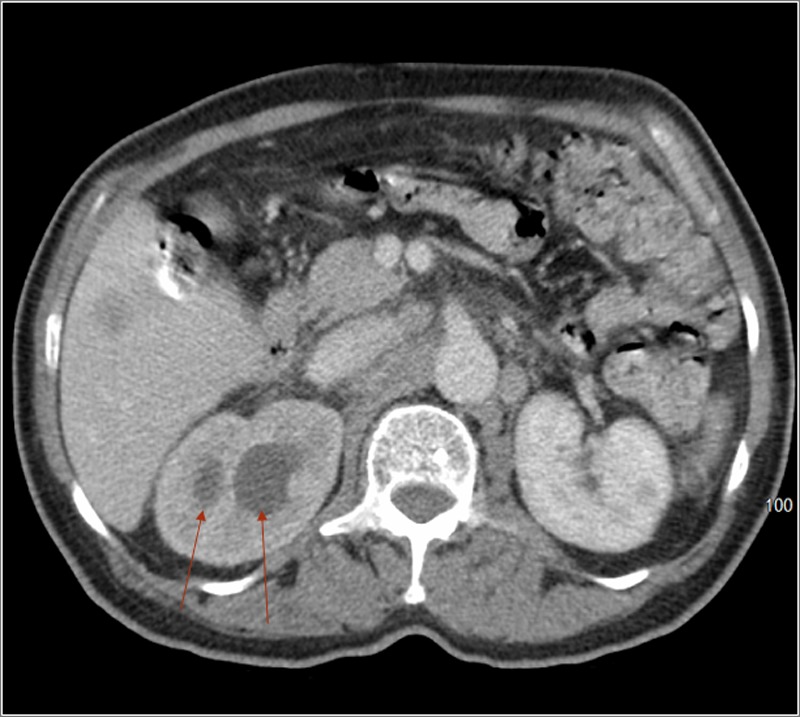
Axial view of CT scan with contrast showing delayed nephrogram and hydronephrosis of both collecting systems (see *arrows*) on the right kidney.

**FIG. 2.**
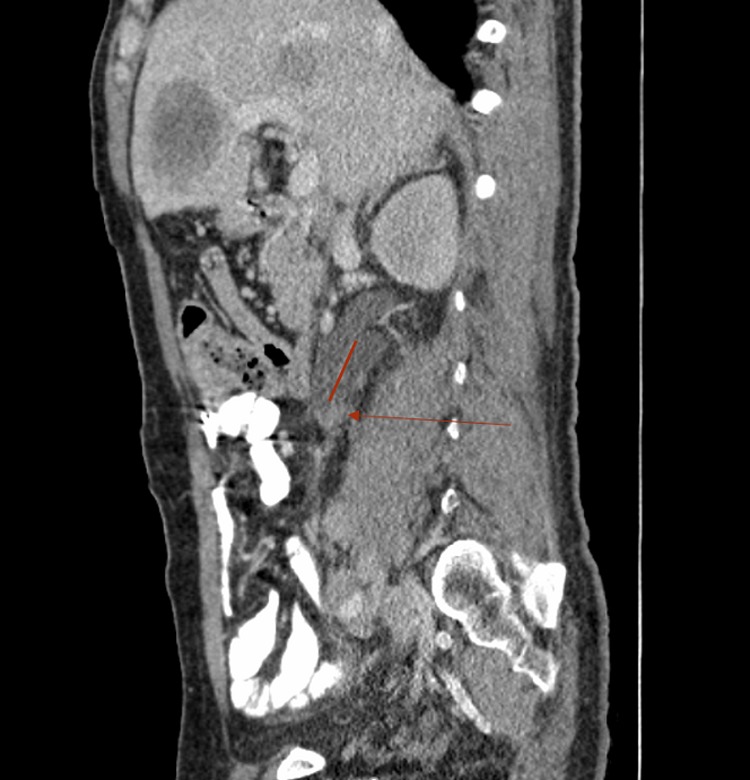
Sagittal view of CT scan with contrast showing a hydronephrotic bifid ureter (*red line* indicating boundary between two ureteral segments) with enhancement at bifurcation (see *arrow*).

Based on systemic symptoms, CT scan was ordered to rule out malignancy. Radiographic findings were remarkable for hypodense liver lesions, pulmonary nodules, retroperitoneal lymphadenopathy, and increased bone density in L1 and L3 vertebral bodies as well as the right iliac bone. The patient also had moderate right hydroureteronephrosis of both renal moieties with delayed nephrogram and transition point within the proximal common right ureter (Figs. 1 and 2). Lumbosacral MRI revealed right L5 nerve root impingement by a lumbar mass. Brain MRI showed abnormal signaling within right temporal lobe concerning for metastasis.

### Intervention

**FIG. 3.**
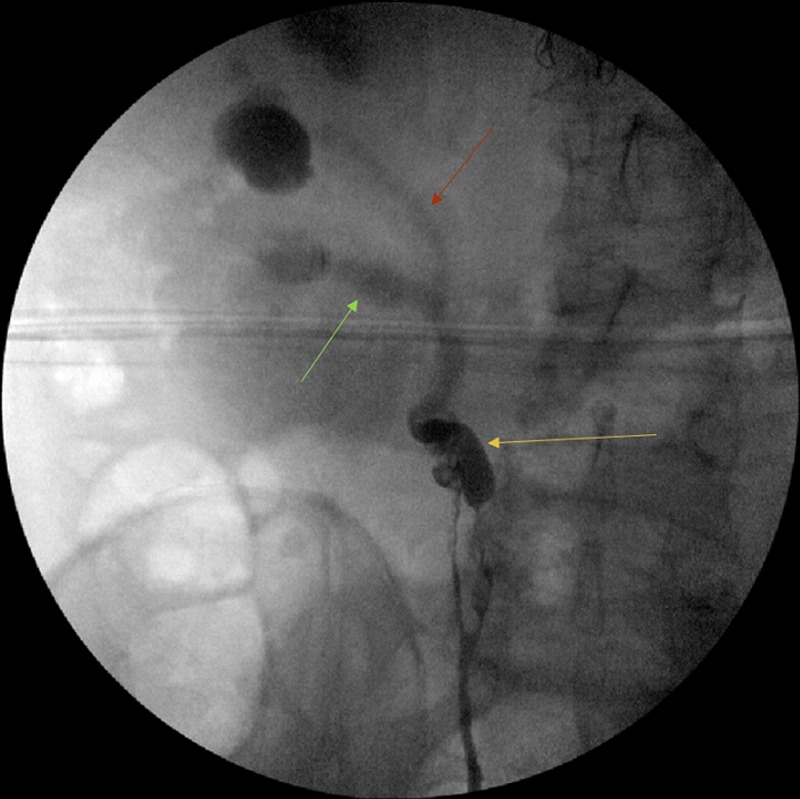
Retrograde pyelogram showing bifurcation at the proximal ureter (*yellow arrow*) with reduced flow through the more proximal ureteral segments (lower pole—*green arrow*, upper pole—*red arrow*) indicating partial obstruction.

To further characterize the ureteral mass as well as to confirm the extent of collecting system duplication, the patient underwent a right ureteroscopy. Cystoscopy revealed bilateral orthotopic ureteral orifices with no ectopic orifice. Right retrograde pyelogram showed a filling defect at the proximal ureter with reduced amount of contrast moving in two levels more proximally, corresponding to a partially duplicated system (Fig. 3). A wire was placed into the upper pole of the kidney. A flexible ureteroscope was advanced alongside the wire confirming a bifurcation of a bifid collecting system at the level of the filling defect. The ureteroscope was advanced into the lower pole ureter, with multiple papillary tumors noted in this segment distally by the bifurcation. Selective barbotage cytology and tissue samples using a Piranha™ grasper (Boston Scientific, Marlborough, MA) were obtained. Narrowing of the proximal right lower pole ureter precluded advancement to the lower pole kidney. Attention was then turned to the upper pole ureter where multiple papillary tumors also were observed at the same level as in the lower pole ureter. Barbotage cytology was again obtained. The upper pole kidney showed no evidence of tumor. Owing to the risk of retrograde seeding of tumor and absence of complete obstruction as demonstrated by adequate emptying films on the retrograde pyelogram, no stent was placed.

### Outcome

Cytology from both right side ureteral moieties returned positive for adenocarcinoma. Tissue biopsy from the right ureter demonstrated metastatic colorectal adenocarcinoma based on immunostaining (e.g., CK20 positive). The patient underwent palliative external beam radiation to the lumbosacral spine and brain followed by initiation of fluorouracil, leucovorin, and oxaliplatin chemotherapy. Although patient's pain improved after starting chemoradiation, his conditioning worsened, eventually becoming bedbound. The patient underwent four cycles of chemotherapy, but interval CT scan showed progression of disease. Ultimately, the patient and family requested termination of chemotherapy and opted for comfort care only.

## Discussion

Stow first reported bilateral ureteral metastasis of an anterior mediastinal lymphosarcoma in 1909.^1^ Since then, ∼400 cases of tumors spreading to the ureter hematogenously or lymphatically as opposed to directly have been described. Breast and intestinal tumors comprise 50% of primary tumors reported; prostate and uterine/cervical cancers account for 30%–40%; and 10% consist of gastric and pancreatic tumors.^2^ Symptoms and signs, when present, as with our patient, are varied and nonspecific; rarely does obstructive uropathy/urologic symptoms present as the first indication of ureteral metastasis. These malignancies are often associated with extensive metastasis at time of diagnosis.^2,3^

Secondary ureteral metastasis can be divided into three categories based on the extent of invasion. Type I, the most common, involves the periureteral adventitial wall only; Type II involves the adventitia and muscle, sparing the mucosa (transmural), and Type III, the least common, involves all three layers. The relative frequency of adventitial involvement is because of prominent periureteral vessels that run parallel to the outer surface of the ureter. Conversely, involvement of the muscle and mucosa only occurs either through direct extension from the adventitia or spread through arterioles branching from the main periureteral arteries.^2,3^ Our case demonstrated a Type III ureteral metastasis from colon cancer, with only four other definite cases published.^4^

Owing to often occult or systemic signs and symptoms, suspicion for ureteral involvement often only occurs after radiographic imaging is obtained. CT will typically show signs of obstructive uropathy (hydronephrosis; delayed nephrogram/excretion of contrast; or filling defects) depending on type of metastasis (e.g., filling defects are typically only seen with Type III lesions).^2^ Of course, even with extensive tumor burden, a patient may still have a coexisting primary ureteral malignancy as opposed to ureteral metastasis. For this reason, ureteroscopy can determine the histology of the malignancy through biopsy and confirm extent of tumor involvement. Only Type III lesions will be seen endoscopically such that tissue biopsy can be obtained. For Type I or Type II lesions not involving the mucosa, fine needle aspiration cytology of the ureteral wall may be performed for diagnosis.^2^ Other diagnostic tests can be performed based on the suspected primary malignancy, such as colonoscopy in our case. Although our patient never received an updated colonoscopy to assess for a primary lesion, his management would not have altered with positive findings.

Regarding treatment, ureteral metastasis is often diagnosed at an advanced stage, with 75% mortality rate within 6 months of diagnosis.^4^ Treatment often consists of chemotherapy with adjunctive therapy (e.g., surgery) based on tumor burden. Surgery can range from curative resection (e.g., nephroureterectomy for an isolated ureteral metastasis biopsy confirmed to be rectal cancer) to palliative relief of ureteral obstruction (i.e., placement of ureteral stent or nephrostomy tube).^4^ Although our patient had partial obstructive uropathy, it did not significantly affect renal function nor was it associated with a complicated urinary tract infection (e.g., pyelonephritis) to mandate urinary decompression.

## Conclusion

We illustrate a rare case of intraluminal ureteral metastasis from colon adenocarcinoma. The metastasis was present in both distal segments of upper and lower ureteral moieties up to the bifurcation of a partially duplicated collecting system, which, to our knowledge, is a pattern of secondary ureteral metastasis that has not been previously described in the literature. Secondary ureteral metastases tend to present with nonspecific symptoms. As such, this case reinforces the importance of diagnostic testing, namely CT imaging to identify the ureteral lesion and adjunctive ureteroscopy to characterize the extent of tumor and confirm true ureteral metastasis through cytology/biopsy. We believe that, in the absence of medical contraindications, ureteroscopy should be performed in all patients that present with a focal ureteral lesion (i.e., absence of any direct extension from adjacent organs) in the setting of metastatic disease to differentiate between primary and secondary ureteral malignancies, given the rarity of the latter. Accurately characterizing a ureteral lesion may have important ramifications in both counseling and treating the patient.

Ultimately, however, despite prompt diagnosis and treatment, many patients succumb to the disease because of advanced stages at presentation. Perhaps with increased use of radiographic imaging, ureteral metastasis may be identified incidentally at earlier stages of disease, which may lead to a more favorable prognosis than currently experienced.^2^

## Abbreviations Used

CT: computed tomography
MRI: magnetic resonance imaging
